# Author Correction: APOE4 exacerbates synapse loss and neurodegeneration in Alzheimer’s disease patient iPSC-derived cerebral organoids

**DOI:** 10.1038/s41467-021-23081-4

**Published:** 2021-05-05

**Authors:** Jing Zhao, Yuan Fu, Yu Yamazaki, Yingxue Ren, Mary D. Davis, Chia-Chen Liu, Wenyan Lu, Xue Wang, Kai Chen, Yesesri Cherukuri, Lin Jia, Yuka A. Martens, Lucy Job, Francis Shue, Thanh Thanh Nguyen, Steven G. Younkin, Neill R. Graff-Radford, Zbigniew K. Wszolek, David A. Brafman, Yan W. Asmann, Nilüfer Ertekin-Taner, Takahisa Kanekiyo, Guojun Bu

**Affiliations:** 1 Department of Neuroscience, Mayo Clinic, Jacksonville, FL USA; 2 Center for Regenerative Medicine, Neuroregeneration Lab, Mayo Clinic, Jacksonville, FL USA; 3 Department of Health Sciences Research, Mayo Clinic, Jacksonville, FL USA; 4 Department of Neurology, Mayo Clinic, Jacksonville, FL USA; 5 School of Biological and Health Systems Engineering, Arizona State University, Tempe, AZ USA

**Keywords:** Mechanisms of disease, Alzheimer's disease

Correction to: *Nature Communications* 10.1038/s41467-020-19264-0, published online 02 November 2020.

The original version of this Article contained an error in Fig. 5b, in which the *y*-axis was incorrectly labelled as ‘ApoE in RIPA’ instead of ‘ApoE in FA’. This has been corrected in both the PDF and HTML versions of the Article.

