# Supplementary material for: APOE4 exacerbates synapse loss and neurodegeneration in Alzheimer’s disease patient iPSC-derived cerebral organoids
Source: Nat Commun. 2020 Nov 2;11:5540. doi: 10.1038/s41467-020-19264-0 (PMC7608683; doi:10.1038/s41467-020-19264-0)
Supplement: Supplementary file 1 — Supplementary Information [file 41467_2020_19264_MOESM1_ESM.pdf]

Supplementary Information

APOE4 exacerbates synapse loss and neurodegeneration in Alzheimer's disease patient iPSC-derived cerebral organoids

Zhao *et al*.

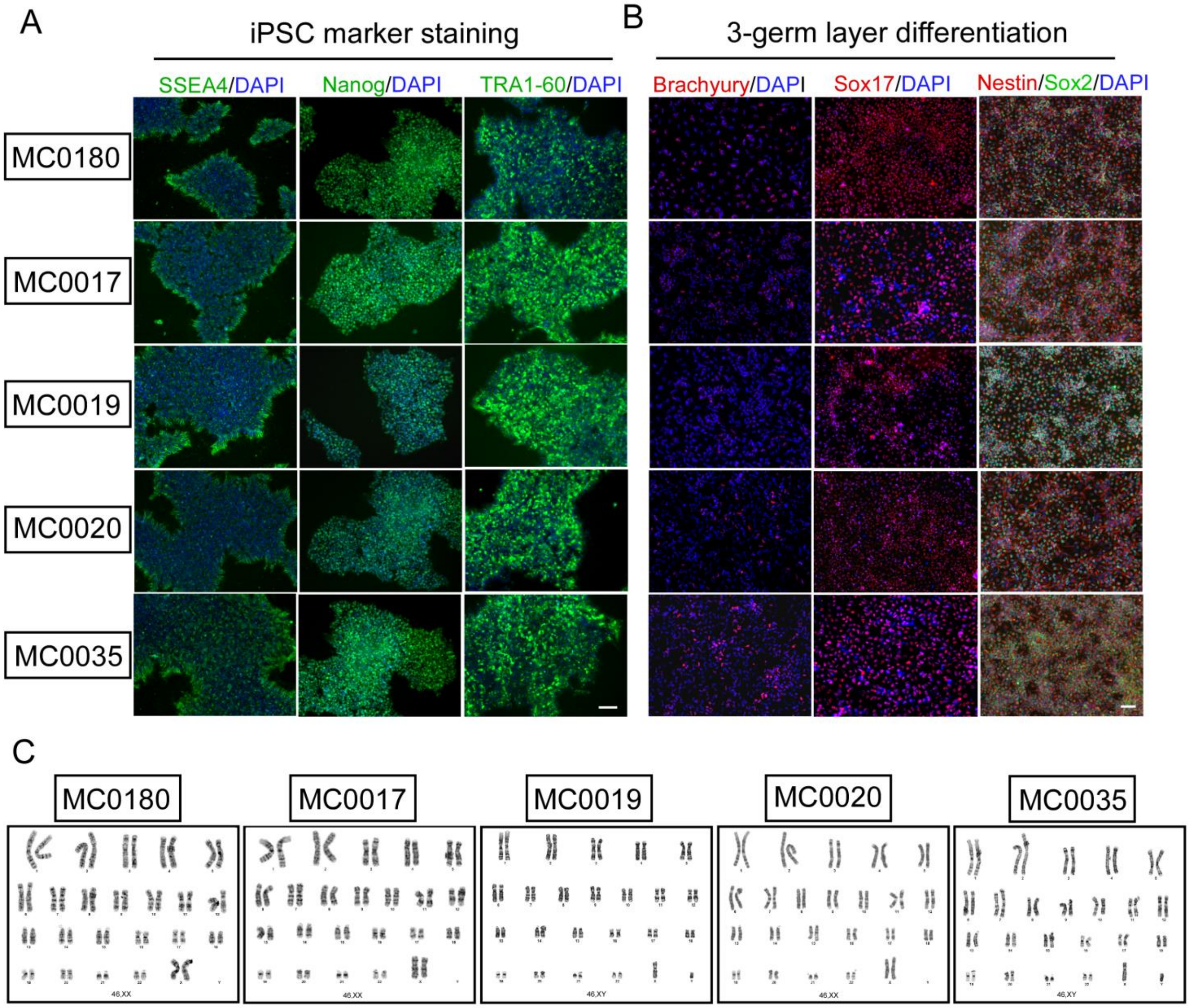

Supplementary Figure 1. Characterization of iPSC lines generated in this study. (A) Immunostaining of pluripotency markers (SSEA4, Nanog, and TRA-1-60) in iPSC lines. (B) In vitro differentiation of iPSC lines into cells of all three germ layers. Cells were immunostained for Brachyury (mesoderm), Sox17 (endoderm), Nestin/Sox2 (ectoderm), and DAPI (nucleus). Scale bar: 100 µm. (C) Karyotyping test confirmed that the number and appearance of chromosomes are normal in iPSC lines.

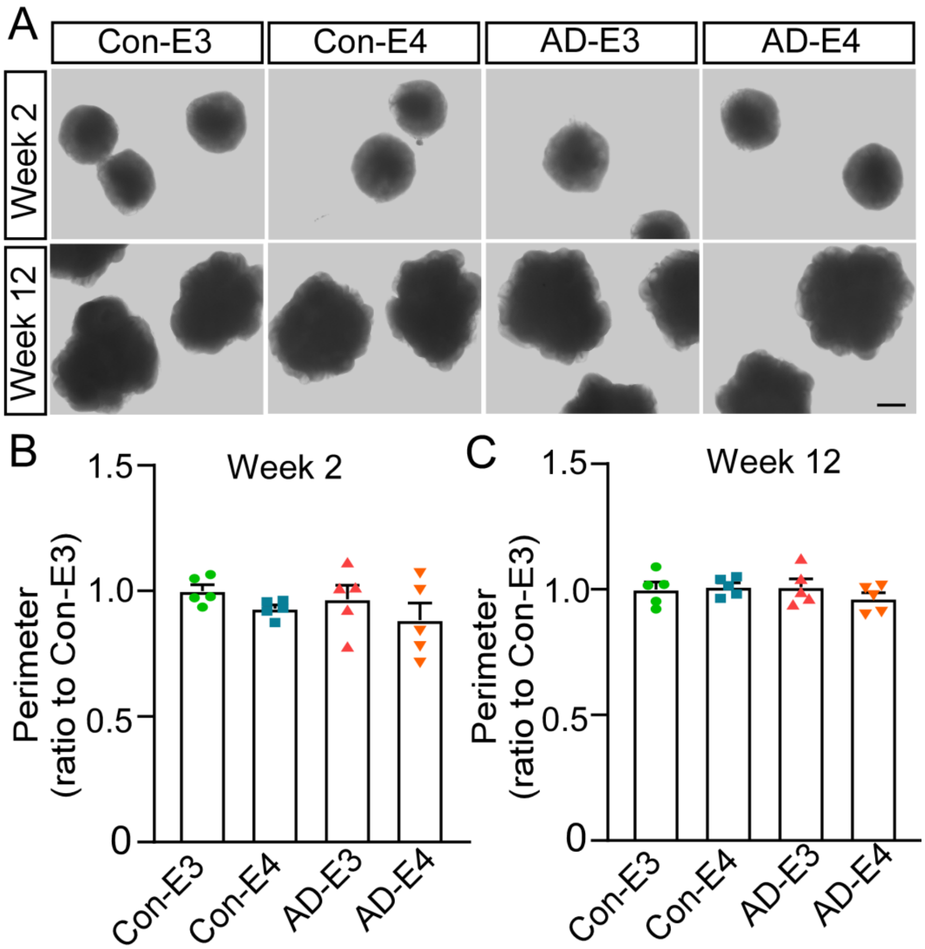

Supplementary Figure 2. Size comparison of iPSC-derived cerebral organoids. (A) Representative images of the cerebral organoids at week 2 and 12. Scale bar: 1 mm. (B-C) Organoid perimeters were quantified in 5 cerebral organoids per line, and the averaged values were compared among groups at week 2 (B; *APOE4:* p=0.1014, AD: p=0.1421, *APOE4* x AD: p=0.6255) and 12 (C; *APOE4:* p=0.2475, AD: p=0.5628, *APOE4* x AD: p=0.1713). All data are expressed as mean ± SEM (N=5). ANCOVA for *APOE4*, AD status, and *APOE4* x AD status was performed by including sex, sampling age, and source of iPSCs as co-variables, which was followed by two-sided Tukey-Kramer tests to compare between the groups with two factors (*APOE4* and AD status).

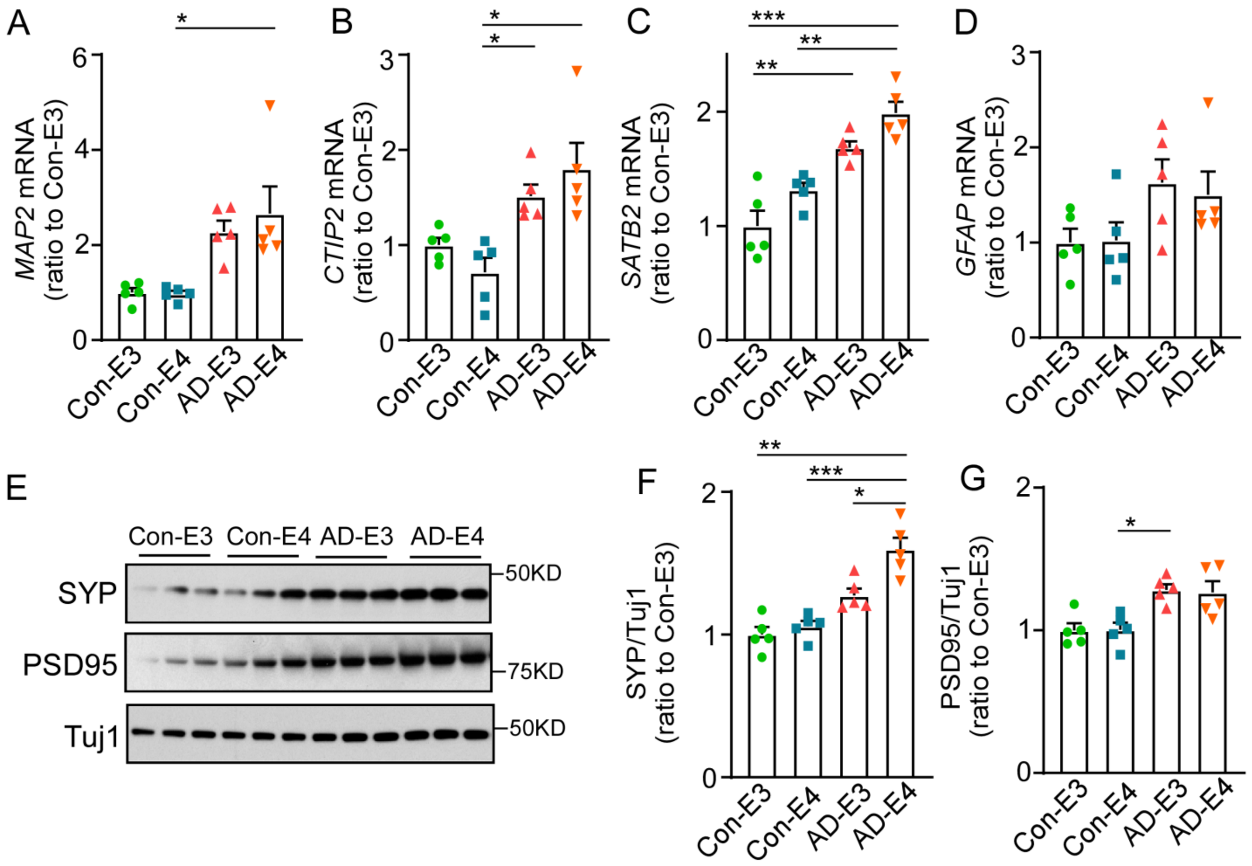

Supplementary Figure 3. Expression of neuronal markers and the levels of synaptic proteins in the cerebral organoids at Week 4. (A-D) The mRNA levels of *MAP2* (A), *CTIP2* (B), *SATB2* (C) and *GFAP* (D) were measure by RT-qPCR in samples of 4-5 cerebral organoids from each line at week 4, and compared among groups (A; *APOE4:* p=0.6725, AD: p=0.0090, *APOE4* x AD: p=0.5480, Con-E4 vs. AD-E4: p=0.0369, B; *APOE4:* p=0.5886, AD: p=0.0061, *APOE4* x AD: p=0.2202, Con-E4 vs. AD-E3: p=0.0342, Con-E4 vs. AD-E4: p=0.0112 C; *APOE4:* p=0.0185, AD: p<0.0001, *APOE4* x AD: p=0.6194, Con-E3 vs. AD-E3: p=0.0053, Con-E3 vs. AD-E4: p=0.0005, Con-E4 vs. AD-E4: p=0.0033 D; *APOE4:* p=0.5716, AD: p=0.0794, *APOE4* x AD: p=0.5531). (E-G) Synaptophysin, PSD95 and Tuj1 levels were analyzed by Western blotting in the lysates of 4-5 cerebral organoids from each line at week 4, and compared among groups. All data are expressed as mean ± SEM (N=5). Synaptophysin and PSD95 levels were normalized to Tuj1 levels, and compared among groups (F; *APOE4:* p=0.0213, AD: p=0.0004, *APOE4* x AD: p=0.0836, Con-E3 vs. AD-E4: p=0.0014, Con-E4 vs. AD-E4: p=0.0006, AD-E3 vs. AD-E4: p=0.0314, G; *APOE4:* p=0.6835, AD: p=0.0065, *APOE4* x AD: p=0.7938, Con-E4 vs. AD-E3: p=0.0424). ANCOVA for *APOE4*, AD status, and *APOE4* x AD status was performed by including sex, sampling age, and source of iPSCs as co-variables, which was followed by two-sided Tukey-Kramer tests to compare between the groups with two factors (*APOE4* and AD status). *p<0.05, **p<0.01, ***p<0.001.

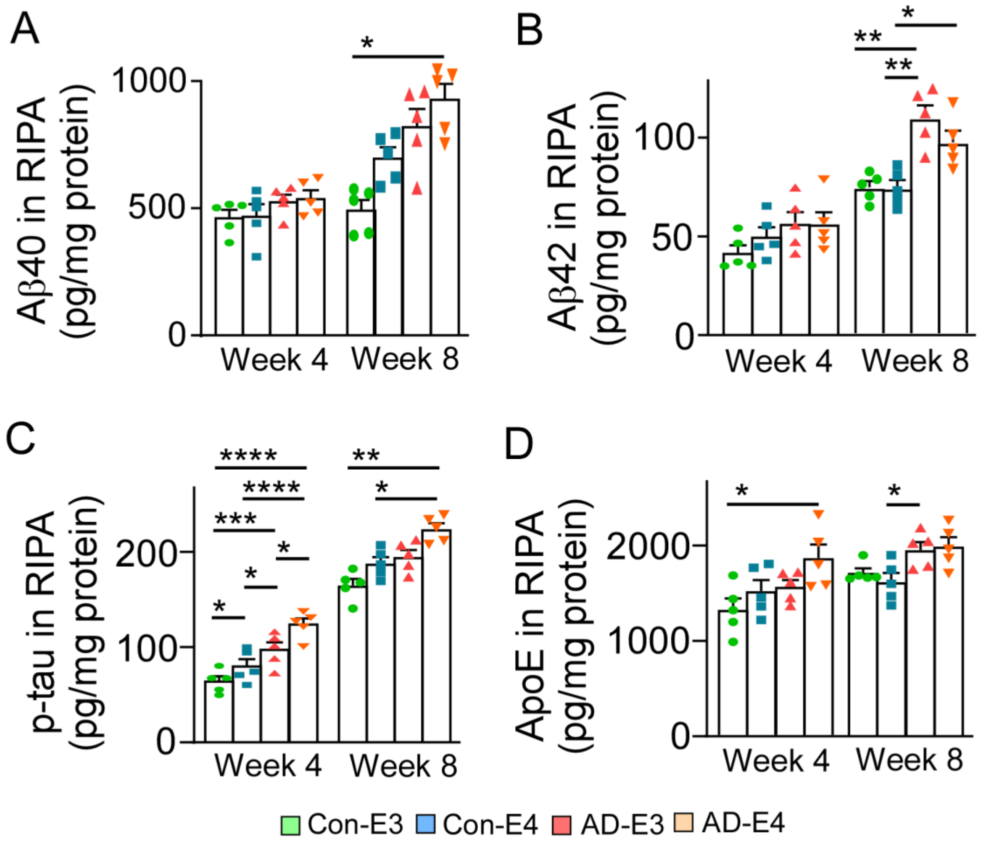

Supplementary Figure 4. The levels of Aβ40, Aβ42, p-tau and apoE in the iPSC-derived cerebral organoids at Week 4 and Week 8. Amounts of Aβ40 (A; Week 4, *APOE4:* p=0.7512, AD: p=0.4544, *APOE4* x AD: p=0.8672; Week 8, *APOE4:* p=0.0276, AD: p=0.0073, *APOE4* x AD: p=0.7106, Con-E3 vs. AD-E4: p=0.0117), Aβ42 (B; Week 4, *APOE4:* p=0.2572, AD: p=0.0218, *APOE4* x AD: p=0.2326; Week 8, *APOE4:* p=0.3519, AD: p=0.0005, *APOE4* x AD: p=0.2394, Con-E3 vs. AD-E3: p=0.0087, Con-E4 vs. AD-E3: p=0.0028, Con-E4 vs. AD-E4: p=0.0399), p-tau (C; Week 4, *APOE4:* p=0.0002, AD: p<0.0001, *APOE4* x AD: p=0.9063, Con-E3 vs. Con-E4: p=0.0181, Con-E3 vs. AD-E3: p=0.0004, Con-E3 vs. AD-E4: p<0.0001, Con-E4 vs. AD-E3: p=0.0241, Con-E4 vs. AD-E4: p<0.0001, AD-E3 vs. AD-E4: p=0.0121; Week 8, *APOE4:* p=0.0044, AD: p=0.0012, *APOE4* x AD: p=0.9781, Con-E3 vs. AD-E4: p=0.0012, Con-E4 vs. AD-E4: p=0.0159) and apoE (D; Week 4, *APOE4:* p=0.0249, AD: p=0.0139, *APOE4* x AD: p=0.9253, Con-E3 vs. AD-E4: p=0.0170; Week 8, *APOE4:* p=0.0801, AD: p=0.0249, *APOE4* x AD: p=0.4763, Con-E4 vs. AD-E3: p=0.0194) in RIPA fractions of 4-5 cerebral organoids per line were measured by ELISA. Data were normalized to individual total protein concentration. All data are expressed as mean ± SEM (N=5). ANCOVA for *APOE4*, AD status, and *APOE4* x AD status was performed by including sex, sampling age, and source of iPSCs as co-variables, which was followed by two-sided Tukey-Kramer tests to compare between the groups with two factors (*APOE4* and AD status). *p<0.05, **p<0.01, ***p<0.001, ****p<0.0001.

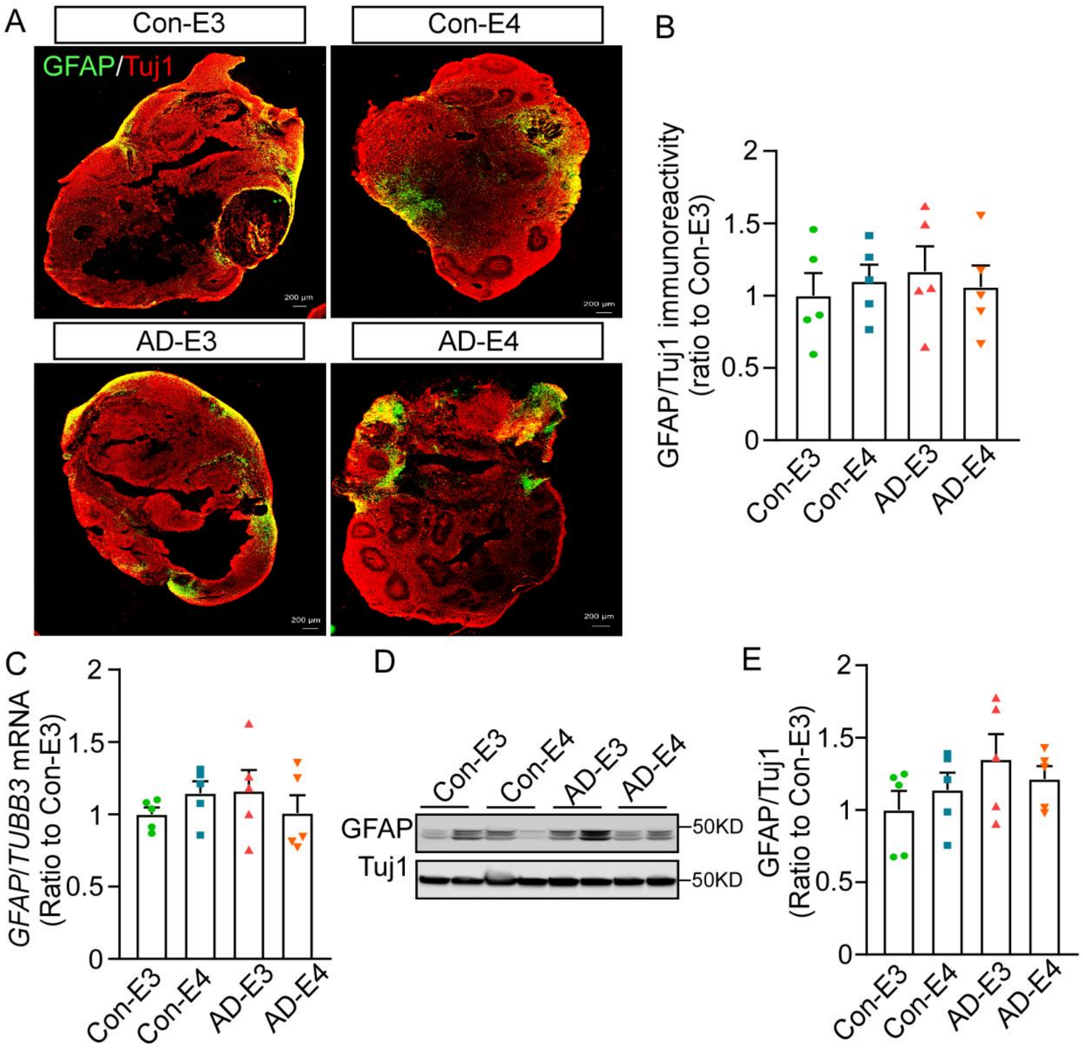

Supplementary Figure 5. GFAP and Tuj1 levels in iPSC-derived cerebral organoids from different groups. Cerebral organoids were subjected to immunostaining, RT-qPCR and Western blotting of GFAP and Tuj1 at week 12. (A) Representative images of immunostaining for GFAP and Tuj1. Scale bar: 200 µm. (B) GFAP/Tuj1 immunoreactivity were quantified and compared among groups (*APOE4*: p=0.8109, AD: p=0.5044, *APOE4* x AD: p=0.5292). (C-E) *GFAP*/*TUBB3* ratio in mRNA levels (C) and GFAP /Tuj1 ratio in protein levels (D, E) were measured by RT-qPCR and Western blotting, respectively, compared among groups (C; *APOE4*: p=0.9009, AD: p=0.8941, *APOE4* x AD: p=0.2748, E; *APOE4*: p=0.9159, AD: p=0.5994, *APOE4* x AD: p=0.6479). All data are expressed as mean ± SEM (N=5). ANCOVA for *APOE4*, AD status, and *APOE4* x AD status was performed by including sex, sampling age, and source of iPSCs as co-variables, which was followed by two-sided Tukey-Kramer tests to compare between the groups with two factors (*APOE4* and AD status).

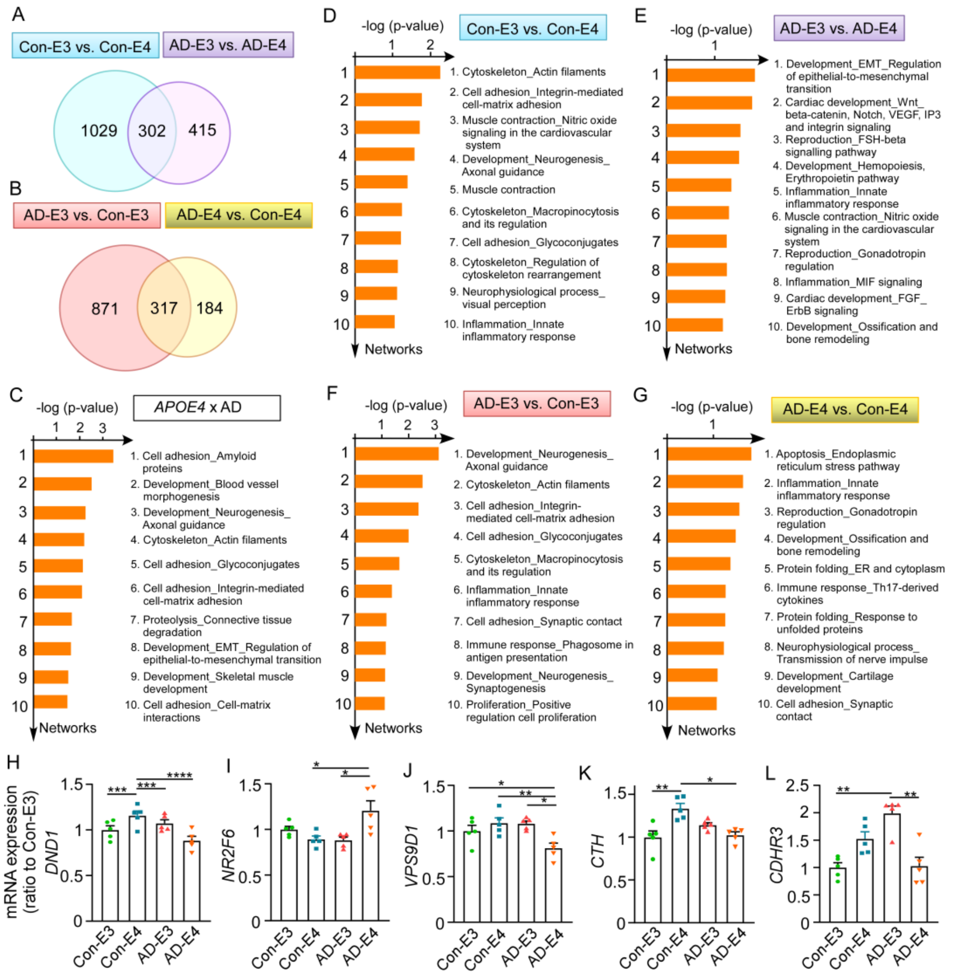

Supplementary Figure 6. DEGs and pathways identified from the RNA-seq data. (A) Overlapped DEGs between Con-E3 vs. Con-E4 and AD-E3 vs. AD-E4. (B) Overlapped DEGs between Con-E3 vs. AD-E3 and Con-E4 vs. AD-E4. (C-G). Top 10 networks enriched by DEGs from *APOE* and disease status interaction (C: p<0.05, |fold change|>2), Con-E3 vs. Con-E4 (D), AD-E3 vs. AD-E4 (E), Con-E3 vs. AD-E3 (F) and Con-E4 vs. AD-E4 (G). (H-L) RT-qPCR validation of selective most differentially expressed genes. The mRNA levels of *DND1* (H; *APOE4*: p= 0.0385, AD: p= 0.0008, *APOE4* x AD: p= 0.0001, Con-E3 vs. Con-E4: p=0.0006, Con-E4 vs. AD-E3: p=0.0008, Con-E4 vs. AD-E4: p<0.0001), *NR2F6* (I; *APOE4*: p= 0.1329, AD: p= 0.1724, *APOE4* x AD: p=0.0169, Con-E4 vs. AD-E4: p=0.0298, AD-E3 vs. AD-E4: p=0.0374), *VPS9D1* (J; *APOE4*: p= 0.0861, AD: p= 0.0137, *APOE4* x AD: p= 0.0284, Con-E3 vs. AD-E4: p=0.0348, Con-E4 vs. AD-E4: p=0.0046, AD-E3 vs. AD-E4: p=0.0386), *CTH* (K; *APOE4*: p= 0.0511, AD: p= 0.7004, *APOE4* x AD: p= 0.0008, Con-E3 vs. Con-E4: p=0.0027, Con-E4 vs. AD-E4: p=0.0199), and *CDHR3* (L; *APOE4*: p= 0.2874, AD: p= 0.1172, *APOE4* x AD: p= 0.0003, Con-E3 vs. AD-E3: p=0.0073, AD-E3 vs. AD-E4: p=0.0044) from 4-5 cerebral organoids per each line were quantified by RT-qPCR at week 12. All data are expressed as mean ± SEM (N=5). ANCOVA for *APOE4*, AD status, and *APOE4* x AD status was performed by including sex, sampling age, and source of iPSCs as co-variables, which was followed by two-sided Tukey-Kramer tests to compare between the groups with two factors (*APOE4* and AD status). *p<0.05, **p<0.01, ***p<0.001, ****p<0.0001.

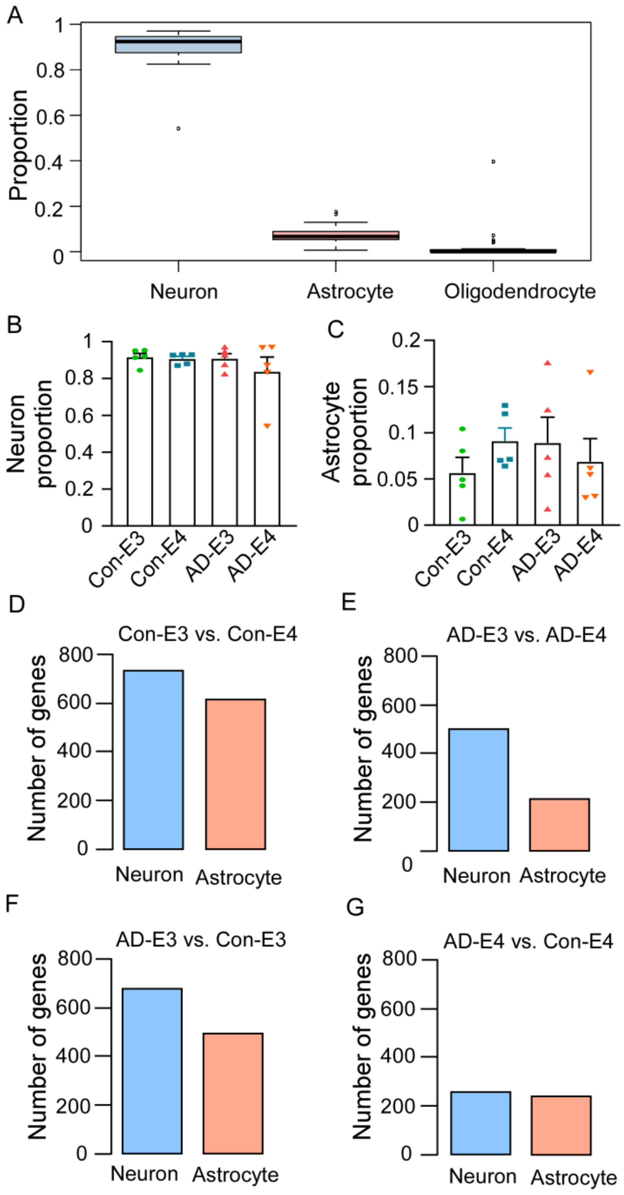

Supplementary Figure 7. Cell composition and DEG assignation to neuron and astrocyte identified from the RNA-seq data. RNA-seq data was analyzed with CIBERSORT program to evaluate the cellular composition in all samples (N=20) (A). The markers represent the minimum, first quartile, median, third quartile, and maximum values, respectively. Outliers identified as 1.5 × the minimum or maximum values are represented by black data points. The proportions of neuron (B) and astrocyte (C) were compared among different groups (mean ± SEM, N=5). ANCOVA for APOE4, AD status, and APOE4 x AD status was performed by including sex, sampling age, and source of iPSCs as co-variables, which was followed by two-sided Tukey-Kramer tests to compare between the groups with two factors (APOE4 and AD status). DEGs assignation to neuron and astrocyte was conducted (D: Con-E3 vs. Con-E4, neuron 722 DEGs, astrocyte 609 DEGs; E: AD-E3 vs. AD-E4, neuron 499 DEGs, astrocyte 218 DEGs; F: AD-E3 vs. Con-E3, neuron 687 DEGs, astrocyte 501DEGs; G: AD-E4 vs. Con-E4, neuron 260 DEGs, astrocyte 241 DEGs).

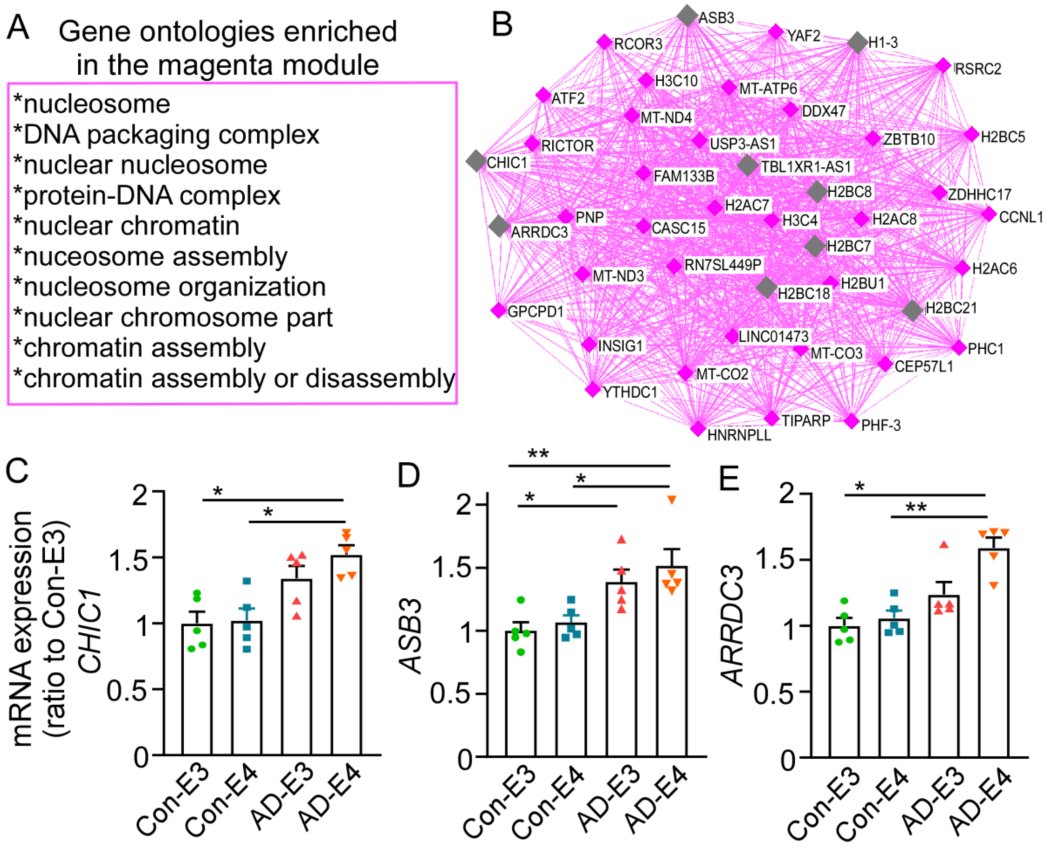

Supplementary Figure 8. Transcriptomics profiling of the iPSC-derived cerebral organoids by WGCNA (magenta module, related to Figure 5). (A) Top gene ontologies enriched by the magenta module genes. (B) Interaction of top 50 genes with the highest connectivity among each other in the magenta module. Gray nodes are hub genes (top 10 highest connectivity), and purple nodes are the rest of the genes. (C-E) Validation of selective hub genes through RT-qPCR. The mRNA expressions of *CHIC1* (C; *APOE4*: p= 0.2512, AD: p= 0.0078, *APOE4* x AD: p= 0.2368, Con-E3 vs. AD-E4: p=0.0476, Con-E4 vs. AD-E4: p=0.0145), *ASB3* (D; *APOE4*: p= 0.1874, AD: p= 0.0008, *APOE4* x AD: 0.8860, Con-E3 vs. AD-E3: p=0.0365, Con-E3 vs. AD-E4: p=0.0083, Con-E4 vs. AD-E4: p=0.0137), and *ARRDC3* (E; *APOE4*: p= 0.0810, AD: p= 0.0070, *APOE4* x AD: p= 0.0708, Con-E3 vs. AD-E4: p=0.0213, Con-E4 vs. AD-E4: p=0.0051), from 4-5 cerebral organoids per each line were quantified by RT-qPCR at week 12. All data are expressed as mean ± SEM (N=5). ANCOVA for *APOE4*, AD status, and *APOE4* x AD status was performed by including sex, sampling age, and source of iPSCs as co-variables, which was followed by two-sided Tukey-Kramer tests to compare between the groups with two factors (*APOE4* and AD status). *p<0.05, **p<0.01.

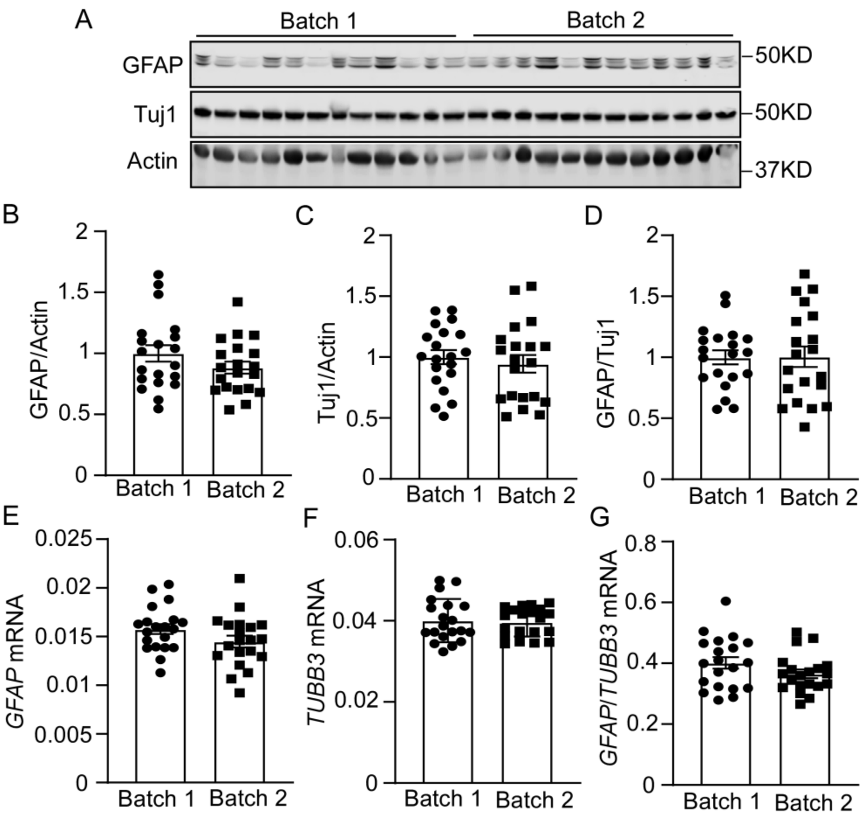

Supplementary Figure 9. Comparison of GFAP and Tuj1 levels in the cerebral organoids prepared during the first and second rounds of differentiation. The levels of GFAP and Tuj1 in the cerebral organoids at week 12 from two batches of differentiation were analyzed and quantified by western blot (A-D) and QPCR (E-G). All data are expressed as mean ± SEM (N=20). Two-sided MannWhitney U tests were performed to determine statistical significance.

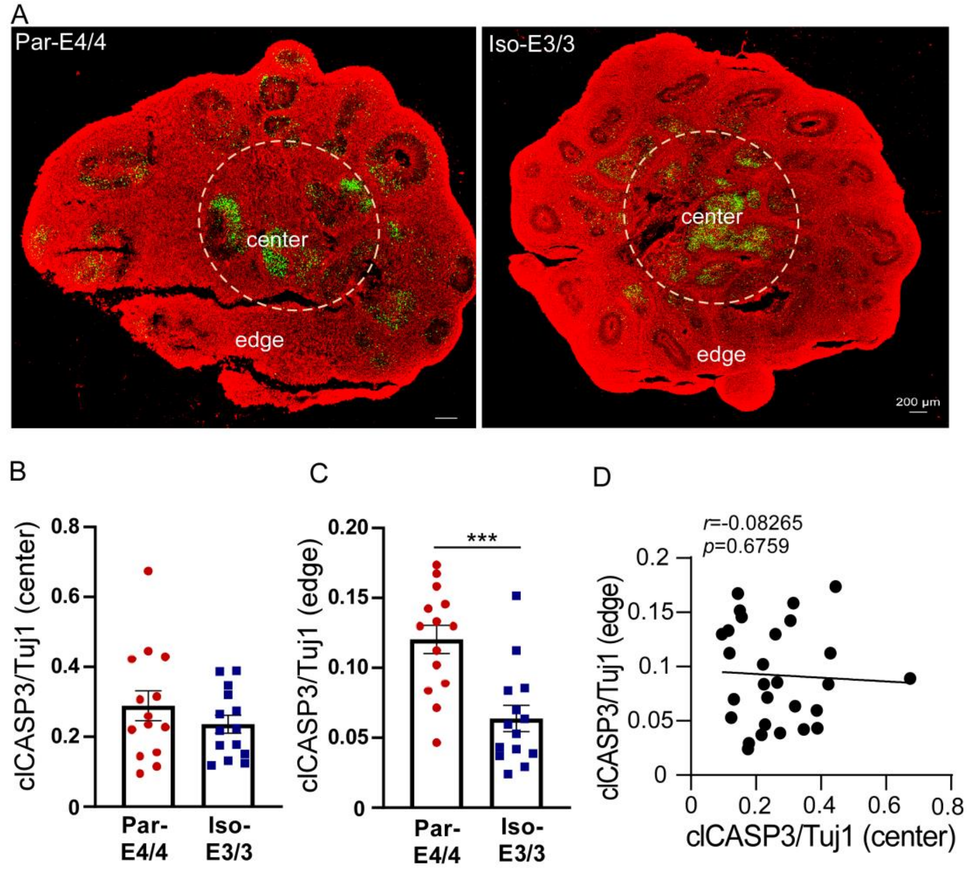

Supplementary Figure 10. Cleaved CASP3 levels at the edge and center areas of cerebral organoids. Cerebral organoids from the AD-*APOE4/4* line and its *APOE3/3* isogenic line were collected for immunostaining of cleaved CASP3 and Tuj1 at Week 12. (A) Representative images of cellular apoptosis evaluated by immunostaining of cleaved CASP3 with Tuj1 counterstaining. Scale bar: 200 µm. (B-C) Quantifications of cleaved CASP3 immunoreactivity normalized by that for Tuj1 at the center (B) and edge of (C: p=0.0006) the cerebral organoids. The center area (1.5-2mm diameter) was subjectively selected in each organoid slice. (D) Two-sided Spearman correlation analysis of the correlation between the cleaved CASP3 level at the center and the edge in cerebral organoids at Week 12. All data are expressed as mean ± SEM (14 slides from 4 organoids were analyzed in each group). Two-sided MannWhitney U tests were performed to determine statistical significance. $^{**}p<0.01$.

Supplementary Table 1

	ID	Sampling age	Sex	*APOE* genotype	Source	Reference
Con E3_1	mc0180	68.3	F	ε3/ε3	Fibroblasts	This report
Con E3_2	mc0017	62.6	F	ε3/ε3	Fibroblasts	This report
Con E3_3	mc0039	72.5	M	ε3/ε3	Fibroblasts	[1]
Con E3_4	mc0192	83	F	ε3/ε3	Fibroblasts	[1]
Con E3_5	mc0117	71	M	ε3/ε3	Fibroblasts	[1]
Con E4_1	mc0116	83	F	ε4/ε4	Fibroblasts	[1]
Con E4_2	mc0115	87	M	ε4/ε4	Fibroblasts	[1]
Con E4_3	mc0018	67.8	F	ε4/ε4	Fibroblasts	[1]
Con E4_4	414-sc8	65	M	ε4/ε4	PBMCs	[2]
Con E4_5	384-sc4	69	F	ε4/ε4	PBMCs	[3]
AD E3_1	mc0019	86.7	M	ε3/ε3	Fibroblasts	This report
AD E3_2	cw50165	78	F	ε3/ε3	PBMCs	CRIM (Fuji)
AD E3_3	cw50120	82	M	ε3/ε3	PBMCs	CRIM (Fuji)
AD E3_4	cw50043	79	F	ε3/ε3	PBMCs	CRIM (Fuji)
AD E3_5	mc0035	77.2	M	ε3/ε3	Fibroblasts	This report
AD E4_1	mc0020	71.3	F	ε4/ε4	Fibroblasts	This report
AD E4_2	cw50104	79	M	ε4/ε4	PBMCs	CRIM (Fuji)
AD E4_3	188-sc18	70	F	ε4/ε4	PBMCs	[2]
AD E4_4	160-sc1	78	F	ε4/ε4	PBMCs	[3]
AD E4_5	cw50129	76	F	ε4/ε4	PBMCs	CRIM (Fuji)
AD-E4/4 (parental)	N/A	64	F	ε4/ε4	Fibroblasts	[4]
AD-E3/3 (isogenic)	N/A	64	F	ε3/ε3	Fibroblasts	[4]

Supplementary References

1. Zhao, J., *et al.* APOE epsilon4/epsilon4 diminishes neurotrophic function of human iPSC-derived astrocytes. *Hum Mol Genet* **26**, 2690-2700 (2017).
2. Brookhouser, N., Zhang, P., Caselli, R., Kim, J.J. & Brafman, D.A. Generation and characterization of two human induced pluripotent stem cell (hiPSC) lines homozygous for the Apolipoprotein e4 (APOE4) risk variant-Alzheimer's disease (ASUi005-A) and healthy non-demented control (ASUi006-A). *Stem Cell Res* **32**, 145-149 (2018).
3. Brookhouser, N., Zhang, P., Caselli, R., Kim, J.J. & Brafman, D.A. Generation and characterization of human induced pluripotent stem cell (hiPSC) lines from an Alzheimer's disease (ASUi001-A) and non-demented control (ASUi002-A) patient homozygous for the Apolipoprotein e4 (APOE4) risk variant. *Stem Cell Res* **24**, 160-163 (2017).
4. Wang, C., *et al.* Gain of toxic apolipoprotein E4 effects in human iPSC-derived neurons is ameliorated by a small-molecule structure corrector. *Nat Med* **24**, 647-657 (2018).
