# Supplementary material for: APOE4 exacerbates synapse loss and neurodegeneration in Alzheimer’s disease patient iPSC-derived cerebral organoids
Source: Nat Commun. 2020 Nov 2;11:5540. doi: 10.1038/s41467-020-19264-0 (PMC7608683; doi:10.1038/s41467-020-19264-0)
Supplement: Supplementary file 3 — Description of Additional Supplementary Files [file 41467_2020_19264_MOESM3_ESM.pdf]

Description of Additional Supplementary Files

Title: Supplementary Data 1

Description: To further address the impact of APOE4 and/or AD status on transcriptional profiles in iPSCderived organoids, we performed RNA-sequencing (RNA-seq) at week 12. Significantly changed genes for different comparison groups are listed in the file. The directions of DEGs are shown as positive (+) or negative (-) fold changes.
