# Supplementary material for: APOE4 exacerbates synapse loss and neurodegeneration in Alzheimer’s disease patient iPSC-derived cerebral organoids
Source: Nat Commun. 2020 Nov 2;11:5540. doi: 10.1038/s41467-020-19264-0 (PMC7608683; doi:10.1038/s41467-020-19264-0)
Supplement: Supplementary file 5 — Reporting Summary [file 41467_2020_19264_MOESM5_ESM.pdf]

nature research

Corresponding author(s): Guojun Bu

Last updated by author(s): 09/17/2020

Reporting Summary

Nature Research wishes to improve the reproducibility of the work that we publish. This form provides structure for consistency and transparency in reporting. For further information on Nature Research policies, see Authors & Referees and the Editorial Policy Checklist .

Statistics

For all statistical analyses, confirm that the following items are present in the figure legend, table legend, main text, or Methods section.

n/a	Confirmed	
☐	☒	The exact sample size (*n*) for each experimental group/condition, given as a discrete number and unit of measurement
☐	☒	A statement on whether measurements were taken from distinct samples or whether the same sample was measured repeatedly
☐	☒	The statistical test(s) used AND whether they are one- or two-sided<br>*Only common tests should be described solely by name; describe more complex techniques in the Methods section.*
☐	☒	A description of all covariates tested
☐	☒	A description of any assumptions or corrections, such as tests of normality and adjustment for multiple comparisons
☐	☒	A full description of the statistical parameters including central tendency (e.g. means) or other basic estimates (e.g. regression coefficient) AND variation (e.g. standard deviation) or associated estimates of uncertainty (e.g. confidence intervals)
☐	☒	For null hypothesis testing, the test statistic (e.g. *F*, *t*, *r*) with confidence intervals, effect sizes, degrees of freedom and *P* value noted<br>*Give P values as exact values whenever suitable.*
☒	☐	For Bayesian analysis, information on the choice of priors and Markov chain Monte Carlo settings
☒	☐	For hierarchical and complex designs, identification of the appropriate level for tests and full reporting of outcomes
☒	☐	Estimates of effect sizes (e.g. Cohen's *d*, Pearson's *r*), indicating how they were calculated

Our web collection on statistics for biologists contains articles on many of the points above.

Software and code

Policy information about availability of computer code

Data collection	Fluorescent signals were detected by fluorescence microscopy (model IX71 Invert, Olympus), confocal laser scanning fluorescent microscopy (model LSM510 Invert, Carl Zeiss), and Keyence fluorescence microscopy (model BZ-X, Keyence). The quantity and quality of all RNA samples for RNA-seq was determined by the Agilent 2100 Bioanalyzer using the Agilent RNA 6000 Nano Chip (Agilent Technologies, CA). QPCR results were collected using an iCycler thermocycler (Bio-Rad). mRNA samples were sequenced at Mayo Clinic using Illumina HiSeq 4000.
Data analysis	Immunocytochemestry signals and western blot results were quantified using ImageJ. The statistical analysis for all experiments was performed using JUMP version 15.0 or GraphPad Prism version 8.0. Detailed analysis was described in each figure legend. MAP-RSeq Version 3.0 and MetaCore software (© MetaCore (Feb 2020) of Clarivate Analytics. All rights reserved) were used for RNA-seq analysis. CIBERSORT analytical tool version 1.0.1, BRETIGEA version 1.0.0 were used for cell composition analysis of RNA-seq data.

For manuscripts utilizing custom algorithms or software that are central to the research but not yet described in published literature, software must be made available to editors/reviewers. We strongly encourage code deposition in a community repository (e.g. GitHub). See the Nature Research guidelines for submitting code & software for further information.

Data

Policy information about availability of data

All manuscripts must include a data availability statement. This statement should provide the following information, where applicable:

- Accession codes, unique identifiers, or web links for publicly available datasets
- A list of figures that have associated raw data
- A description of any restrictions on data availability

Full scans of the gels and blots are available in Source data file. All relevant data are available from the corresponding author upon reasonable request. All the other data supporting the findings of this study are available within the article and its supplementary information files and from the corresponding author upon reasonable request. A reporting summary for this article is available as a Supplementary Information file.

The RNA-seq data are available via the AD Knowledge Portal [https://adknowledgeportal.synapse.org]. The AD Knowledge Portal is a platform for accessing data, analyses, and tools generated by the Accelerating Medicines Partnership (AMP-AD) Target Discovery Program and other National Institute on Aging (NIA)-supported programs to enable open-science practices and accelerate translational learning. The data, analyses and tools are shared early in the research cycle without a publication embargo on secondary use. Data is available for general research use according to the following requirements for data access and data attribution [https://adknowledgeportal.synapse.org/DataAccess/Instructions]. For access to content described in this manuscript see: [https://doi.org/10.7303/syn22307008].

Field-specific reporting

Please select the one below that is the best fit for your research. If you are not sure, read the appropriate sections before making your selection.

☒ Life sciences ☐ Behavioural & social sciences ☐ Ecological, evolutionary & environmental sciences

For a reference copy of the document with all sections, see nature.com/documents/nr-reporting-summary-flat.pdf

Life sciences study design

All studies must disclose on these points even when the disclosure is negative.

Sample size	The number of iPSC lines and controls proposed were determined based on the current standards of iPSC work established by the International Society for Stem Cell Research (Guidelines for Stem Cell Science and Clinical Translation, 2016; available at http://www.isscr.org/guidelines2016) and previously published literature. In the first paper on modeling Alzheimer's disease using iPSC-derived neurons by Yagi et al. in 2011, they generated iPSC lines from two AD patients and two healthy control, two clones for each line were used in the experiments. In another paper by Muratore et al, 2014, they collected 4 iPSC lines from healthy control, 2 iPSC lines from AD patients with two colonies for each. Based on these publications, we decided to use 5 iPSC lines for each group.
Data exclusions	No cell line samples were excluded from analyses.
Replication	Same experiments were replicated in second batch of cerebral organoid culture. All replications were consistent.
Randomization	Samples were allocated in different experimental groups based on their APOE genotypes and AD disease status.
Blinding	Blinding was not relevant to the study because all cell lines are culture in the same condition, and collected and analysis in the same methods. The investigators who performed the quantification of the immunostainings were blinded to the genotype of the samples

Reporting for specific materials, systems and methods

We require information from authors about some types of materials, experimental systems and methods used in many studies. Here, indicate whether each material, system or method listed is relevant to your study. If you are not sure if a list item applies to your research, read the appropriate section before selecting a response.

Materials & experimental systems

n/a	Involved in the study	
☐	☒	Antibodies
☐	☒	Eukaryotic cell lines
☒	☐	Palaeontology
☒	☐	Animals and other organisms
☐	☒	Human research participants
☒	☐	Clinical data

Methods

n/a	Involved in the study	
☒	☐	ChIP-seq
☒	☐	Flow cytometry
☒	☐	MRI-based neuroimaging

Antibodies

Antibodies used	Immunostaining:<br>Nanog (Cell Signaling, 4903, 1 : 300), TRA-1-60 (Abcam, ab16288, 1 : 300), SSEA4 (Abcam, ab16287, 1 : 300), Sox17 (Abcam, ab84990, 1 : 300), Brachyury (R&D, AF2085, 1 : 300), Nestin (Abcam, ab18102, 1:500), Sox2 (Abcam, ab97959, 1:500), TUJ1 (Abcam, ab78078, 1:1000), TUJ1 (Sigma, T2200, 1:1000), CTIP2 (Abcam, ab18465, 1:100), SATB2 (Abcam, ab34735, 1:100), GFAP (Millipore, MAB360, 1:300), cleaved Caspase-3 (Cell Signaling Technology, 9579, 1:300), AT8 (Thermo Fisher Scientific, MN1020, 1:300), G3BP (BD, 611126, 1:300). Alexa Fluor donkey anti-rabbit 488, 594 (Invitrogen, A32790, A32754, 1:500), Alexa Fluor donkey anti-mouse 488, 594 (Invitrogen A32766, A32744, 1:500), and Alexa Fluor donkey anti-rat 488, 594 (Invitrogen A-21208, A-21209, 1:500).<br>Western blots:<br>Cleaved Caspase-3 (Cell Signaling Technology, 9661, 1:1000), Caspase-3 (Cell Signaling Technology, 9662, 1:1000), PSD95 (Abcam, ab2723, 1 : 1000), Synaptophysin (Abcam, ab8049, 1:1000), AT8 (Thermo Fisher Scientific, MN1020, 1:1000), PHF-1 (Abcam, ab184951, 1:1000), Tau5 (Millipore, 577801, 1:1000), APP (Thermo Fisher Scientific, 14-9749-82, 1:1000) EEA1 (Cell

Signaling Technology, 2411, 1:1000), LAMP1 (Cell Signaling Technology, 9091, 1:1000), G3BP (BD, 611126, 1:1000), GFAP (Millipore, MAB360, 1:1000), ERCC4 (Fitzgerald, 10R-4026, 1:1000), POLR3A (Abcam, ab96328, 1:1000) and HSPA4 (Cell Signaling Technology, 3303, 1:1000).

Validation

Antibodies used in our study have been highly cited in the filed and validated by multiple lab. We have focused on the citation lists as validation to support our claim. According to the manufacturer's website:
The Cleaved Caspase 3 antibody (Cell Signaling Technologies, 9661) is reactive to human tissue, and has been cited in 3,488 publications for both immunostaining and western blots, including Velasco et al., 2019, which used this antibody on human brain organoids.
The CTIP2 antibody (Abcam, ab18465) is reactive to human tissue, and has been cited in 295 publications, including Quadrato et al., 2017, which used this antibody on human brain organoids.
The Nanog antibody (Cell Signaling, 4903) is reactive to human tissue, and has been cited in 159 publications, including Zhao et al., 2017, from our lab, which used this antibody for iPSC characterization.
The TRA-1-60 antibody (Abcam, ab16288) is reactive to human tissue, and has been cited in 74 publications, including Zhao et al., 2017, from our lab, which used this antibody for iPSC characterization.
The SSEA4 antibody (Abcam, ab16287) is reactive to human tissue, has been cited in 114 publications, including Zhao et al., 2017, from our lab, which used this antibody for iPSC characterization.
The Sox17 antibody (Abcam, ab84990) is reactive to human tissue, and has been cited in 11 publications.
The Brachyury antibody (R&D, AF2085) is reactive to human tissue, and has been cited in 68 publications.
The Nestin antibody (Abcam, ab18102) is reactive to human tissue, has been cited in 9 publications.
The Sox2 antibody (Abcam, ab97959) is reactive to human tissue, has been cited in 358 publications, Ballabio C et al. 2020, which used this antibody for cerebral organoid.
The TUJ1 antibody (Abcam, ab78078) is reactive to human tissue, has been cited in 110 publications.
The TUJ1 antibody (Sigma, T2200) is reactive to human tissue, has been cited in 291 publications.
The SATB2 antibody (Abcam, ab34735) is reactive to human tissue, has been cited in 43 publications.
The GFAP antibody (Millipore, MAB360) is reactive to human tissue, has been cited in 384 publications for both immunostaining and western blots.
The AT8 antibody (Thermo Fisher Scientific, MN1020) is reactive to human tissue, has been cited in 600 publications for both immunostaining and western blots.
The G3BP antibody (BD, 611126) is reactive to human tissue, has been cited in 2 publications.
The Caspase-3 antibody (Cell Signaling Technology, 9662) is reactive to human tissue, has been cited in 2240 publications.
The PSD95 antibody (Abcam, ab2723) is reactive to human tissue, has been cited in 106 publications.
The Synaptophysin antibody (Abcam, ab8049) is reactive to human tissue, has been cited in 109 publications.
The PHF-1 antibody (Abcam, ab184951, 1:1000) is reactive to human tissue, has been cited in 9 publications.
The Tau5 antibody (Millipore, 577801) is reactive to human tissue, has been cited in 5 publications.
The APP antibody (Thermo Fisher Scientific, 14-9749-82) is reactive to human tissue. Expression of APP was observed in in Mouse and Rat Brain, but not in the mouse and rat skeletal muscle in western blots.
The EEA1 antibody (Cell Signaling Technology, 2411) is reactive to human tissue, has been cited in 73 publications.
The LAMP1 antibody (Cell Signaling Technology, 9091) is reactive to human tissue, has been cited in 149 publications.
The ERCC4 antibody (Fitzgerald, 10R-4026) is reactive to human tissue. In the website, western blots show bands in HEK293T cell lysates transfected with recombinant ERCC4 protein, but not in the empty vector group.
The POLR3A antibody (Abcam, ab96328) is reactive to human tissue, has been cited in 8 publications.
The HSPA4 (Cell Signaling Technology, 3303) is reactive to human tissue, has been cited in 11 publications.
The Cleaved Caspase 3 antibody (Cell Signaling Technologies, 9661) is reactive to human tissue, and has been cited in 3,488 publications for both immunostaining and western blots, including Velasco et al., 2019, which used this antibody on human brain organoids.
The CTIP2 antibody (Abcam, ab18465) is reactive to human tissue, and has been cited in 295 publications, including Quadrato et al., 2017, which used this antibody on human brain organoids.
The Nanog antibody (Cell Signaling, 4903) is reactive to human tissue, and has been cited in 159 publications, including Zhao et al., 2017, from our lab, which used this antibody for iPSC characterization.
The TRA-1-60 antibody (Abcam, ab16288) is reactive to human tissue, and has been cited in 74 publications, including Zhao et al., 2017, from our lab, which used this antibody for iPSC characterization.
The SSEA4 antibody (Abcam, ab16287) is reactive to human tissue, has been cited in 114 publications, including Zhao et al., 2017, from our lab, which used this antibody for iPSC characterization.
The Sox17 antibody (Abcam, ab84990) is reactive to human tissue, and has been cited in 11 publications.
The Brachyury antibody (R&D, AF2085) is reactive to human tissue, and has been cited in 68 publications.
The Nestin antibody (Abcam, ab18102) is reactive to human tissue, has been cited in 9 publications.
The Sox2 antibody (Abcam, ab97959) is reactive to human tissue, has been cited in 358 publications, Ballabio C et al. 2020, which used this antibody for cerebral organoid.
The TUJ1 antibody (Abcam, ab78078) is reactive to human tissue, has been cited in 110 publications.
The TUJ1 antibody (Sigma, T2200) is reactive to human tissue, has been cited in 291 publications.
The SATB2 antibody (Abcam, ab34735) is reactive to human tissue, has been cited in 43 publications.
The GFAP antibody (Millipore, MAB360) is reactive to human tissue, has been cited in 384 publications for both immunostaining and western blots.
The AT8 antibody (Thermo Fisher Scientific, MN1020) is reactive to human tissue, has been cited in 600 publications for both immunostaining and western blots.
The G3BP antibody (BD, 611126) is reactive to human tissue, has been cited in 2 publications.
The Caspase-3 antibody (Cell Signaling Technology, 9662) is reactive to human tissue, has been cited in 2240 publications.

	The PSD95 antibody (Abcam, ab2723) is reactive to human tissue, has been cited in 106 publications.<br>The Synaptophysin antibody (Abcam, ab8049) is reactive to human tissue, has been cited in 109 publications.<br>The PHF-1 antibody (Abcam, ab184951, 1:1000) is reactive to human tissue, has been cited in 9 publications.<br>The Tau5 antibody (Millipore, 577801) is reactive to human tissue, has been cited in 5 publications.<br>The APP antibody (Thermo Fisher Scientific, 14-9749-82) is reactive to human tissue. Expression of APP was observed in in Mouse and Rat Brain, but not in the mouse and rat skeletal muscle in western blots.<br>The EEA1 antibody (Cell Signaling Technology, 2411) is reactive to human tissue, has been cited in 73 publications.<br>The LAMP1 antibody (Cell Signaling Technology, 9091) is reactive to human tissue, has been cited in 149 publications.<br>The ERCC4 antibody (Fitzgerald, 10R-4026) is reactive to human tissue. In the website, western blots show bands in HEK293T cell lysates transfected with recombinant ERCC4 protein, but not in the empty vector group.<br>The POLR3A antibody (Abcam, ab96328) is reactive to human tissue, has been cited in 8 publications.<br>The HSPA4 (Cell Signaling Technology, 3303) is reactive to human tissue, has been cited in 11 publications.

Eukaryotic cell lines

Policy information about cell lines

Cell line source(s)	The sources of cell lines were listed in Table 1 in the manuscript
Authentication	Cell lines were confirmed to have normal karyotypes after reprogramming. APOE genotype were confirmed by TaqMan SNP Genotyping. The pluripotency of the iPSC lines were also validated by their ability to differentiate into three germ layers.
Mycoplasma contamination	All iPSC lines were were tested negative for mycoplasma contamination.
Commonly misidentified lines<br>(See ICLAC register)	No cell lines used in the study are listed in the ICLAC database of misidentified cell lines.

Human research participants

Policy information about studies involving human research participants

Population characteristics	Skin biopsies from healthy subjects and AD patients were collected with the agreement from each individuals. Fibroblasts were generated from the skin biopsies, characterized for APOE genotype and further stocked in the cell bank of Neuroregeneration Lab(NRL) in Mayo Clinic, Jacksonville. Fibroblasts with matching age, gender and APOE genotype were requested from the NRL and used in the current study.
Recruitment	The study participants were recruited by neurologists in Mayo Clinic without selection bias. Written consent was obtained from the participants before skin biopsy.
Ethics oversight	The study was approved by the Mayo Clinic Institutional Review Board (IRB 09-00380301 and 12-002562). IRB 09-00380301 is for collecting skin biopsy, and the IRB 12-002562 is for actually using the fibroblast and iPSCs for research.

Note that full information on the approval of the study protocol must also be provided in the manuscript.
